# Thyroid function test evolution in children with Hashimoto’s thyroiditis is closely conditioned by the biochemical picture at diagnosis

**DOI:** 10.1186/s13052-018-0461-5

**Published:** 2018-02-07

**Authors:** Giuseppe Crisafulli, Romina Gallizzi, Tommaso Aversa, Giuseppina Salzano, Mariella Valenzise, Malgorzata Wasniewska, Filippo De Luca, Giuseppina Zirilli

**Affiliations:** Department of Human Pathology in Adulthood and Childhood, University of Messina, Via Consolare Valeria, 98124 Messina, Italy

**Keywords:** Hashitoxicosis, Overt hyperthyroidism, Overt hypothyroidism, Subclinical hyperthyroidism, Subclinical hypothyroidism, Thyroid status natural course

## Abstract

**ᅟ:**

Aim of this commentary is to summarize the salient literature views on the relationships between presentation and evolution patterns of thyroid function in children with Hashimoto’s thyroiditis (HT).

According to the most recent reports, children with HT and subclinical hypothyroidism (SH) are more prone to the risk of developing severe thyroid dysfunctions over time, if compared to those presenting with euthyroidism.

In contrast, children presenting with HT and either overt or subclinical hyperthyroidism are incline to exhibit a definitive resolution of the hyperthyroid phase within some months, although there is a wide variability between the different individuals.

The natural history of frank hypothyroidism in the children with HT has never been investigated so far, since in these cases an immediate onset of replacement treatment is mandatory.

**Conclusions:**

1) a deterioration of thyroid status over time may be observed especially in the children presenting with SH, but also in those presenting with euthyroidism; 2) a definitive resolution of the hyperthyroid phase is generally observed in those presenting with either overt or subclinical hyperthyroidism.

## Background

Hashimoto’s thyroiditis (HT) is a relatively common disease, whose prevalence in childhood has been reported to range around 3% and to achieve its peak during adolescence [1]. It is the commonest form of thyroiditis in pediatric age [2] and the most frequent cause of pediatric thyroid disease in iodine – replete areas of the world [3].

At the time of diagnosis, children with HT are frequently asymptomatic, with a thyroid function picture ranging from euthyroidism (52.1% of cases) to either overt hypothyroidism (in 22.2% of cases) or, more rarely, subclinical hypothyroidism (SH) (in 19.2% of cases) [4]. Other complaints of thyroid function, which may be sometimes (6.5% of cases) encountered in pediatric age at HT presentation, include either subclinical or overt hyperthyroidism [4–6]. It has been also reported that, in at least 3–4% of the children with Graves’ disease (GD), the onset of hyperthyroidism may be preceded by HT antecedents [7], which suggests the existence of a continuum between HT and GD, within the broad spectrum of autoimmune thyroid disorders (AITDs) [8–10].

According to the available pediatric epidemiological studies, the prevalence rates of thyroid function patterns, at HT diagnosis, may significantly vary in the different series [4, 11–17], as summarized in Table 1.

**Table 1:** Prevalence rates (%) of the main thyroid function patterns, at Hashimoto’s thyroiditis presentation, in pediatric cohorts, according to different epidemiological studies

Authors	Nos. patients	Euthyroidism	Subclinical hypothyroidism	Overt hypothyroidism	Hyperthyroidism
Zak et al. (2005) [11]	100	63	26	─	11
Svensson et al. (2006) [12]	90	39	47	14	─
Demirbilek et al. (2007) [13]	162	43.2	24.1	21	not stated
Gopalakrishnan et al. (2007) [14]	98	24.5	32.6	42.9	─
de Vries et al. (2009) [15]	114	21	42	37	─
Ozen et al. (2011) [16]	101	36.7	32.7	16.8	13.8
Skarpa et al. (2011) [17]	228	57	32.9	8.3	1.8
Wasniewska et al. (2012) [4]	608	52.1	19.2	22.2	6.5

Although the recent literature includes many reports on the biochemical presentation of HT in pediatric age [4–6, 11–17], only few studies have specifically addressed whether the hormonal pattern at HT presentation may in any way condition the subsequent course of thyroid function tests over time [5, 18–20].

The aim of the present review is to analyze the available studies on this topic, i.e. the possible relationships between presentation and evolution patterns of thyroid function in children with HT.

### Long-term prognosis of thyroid function in the cases presenting with euthyroidism

Euthyroidism is the commonest presentation pattern of HT in pediatric age [4–6].

Whereas in adults it is often observed a progressive shift from euthyroidism toward SH or frank hypothyroidism [21], in childhood and adolescence the natural long-term evolution of thyroid tests may be quite variable. In fact, in the 4-year follow-up study by Radetti et al. [18], a large majority of initially euthyroid patients remained euthyroid. In contrast, according to the more recent study by Aversa et al. [20], only 57.1% of initially euthyroid children remained euthyroid even five years after HT diagnosis. The remaining 42.9% deteriorated their thyroid status over time, thus developing a SH in 30.6% of cases and an overt biochemical hypothyroidism in 12.3% [20]. The presence of goiter and elevated thyroglobulin autoantibodies at HT diagnosis might be considered as predictor for the future development of hypothyroidism [18].

On overall, from the analysis of the available longitudinal studies concerning the long-term prognosis of thyroid function in patients with HT, it may be argued that even the children who are initially euthyroid, at the time of HT diagnosis, should undergo a biochemical follow-up of thyroid function. A periodical monitoring of thyroid tests over time is even more indicated in adults, who are more incline than young patients to the risk of deteriorating their thyroid status [21].

### Long-term prognosis of thyroid function in the cases presenting with SH

**Fig. 1:**
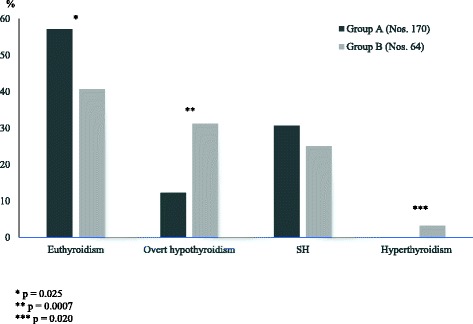
Prevalence (%) of the different biochemical pictures of thyroid function found, at the end of a 5-year follow-up, in two groups of children with Hashimoto’s thyroiditis, who had initially presented with either euthyroidism (Group A) or subclinical hypothyroidism SH (Group B) (according to the results of Reference 20 study)

The natural evolution of thyroid status, in HT children who presented with SH, has been just recently reported to be more severe than in those who presented with euthyroidism [20]. In fact, at the end of a 5-year follow-up, the prevalence of patients with overt hypothyroidism was significantly higher in the cohort with initial SH, whereas the prevalence of those with euthyroidism was significantly higher in the other group (Fig. 1). Furthermore, in the same study [20], a 0.8% of the children who had presented with SH developed over time a picture of GD (Fig. 1), a sequence of events which has been reported to occur more often in young patients with either Turner syndrome (TS) or Down syndrome (DS) [22–27], but may also be observed in the general pediatric population [7–10].

It has to be underlined that the long-term prognosis of thyroid status in children with HT-related SH is not necessarily unfavorable, since 40.6% of the SH patients included in the study by Aversa et al. [20] spontaneously normalized TSH values at the end of follow-up (Fig. 1), as also observed by other authors [28–30].

Thyroid function prognosis, however, is on overall significantly more severe in children with HT-related SH than in those with idiopathic SH and underlying AITDs [31]. In fact, the percentage of SH children who, during a 2-year follow-up, increase TSH values to > 10 mU/l and require L-T4 therapy is known to be significantly higher in the subgroup with HT-related SH than in those with idiopathic SH [31]. Therefore, it may be inferred that the association with HT is able to exert a negative impact on the course of SH in pediatric age.

It has to be emphasized that the process of spontaneous deterioration of thyroid function, that may occur over time in the children with HT-related SH, may be very slow and not predictable in the single case [19]. Therefore, although surveillance is mandatory in all these cases, it might be necessary a long time to infer whether L-T4 treatment should be implemented or not [20]. The coexistence of additional risk factors, such as celiac disease or elevated TSH and thyroid peroxidase autoantibodies, at HT diagnosis, seems to augment the probabilities that SH children with HT may develop a frank hypothyroidism 3 years later [19]. Thus, an elevated TSH at HT diagnosis could be considered as the best predictor for a thyroid function deterioration from SH to overt hypothyroidism, as already suggested by other studies concerning patients with SH and no underlying AITDs [32, 33].

**Fig. 2:**
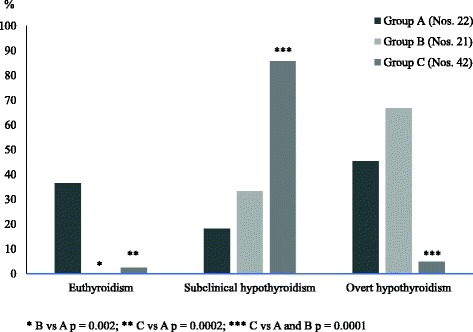
Prevalence (%) of the main biochemical pictures of thyroid function found, at the end of a 5-year follow-up, in three groups of children with Hashimoto’s thyroiditis (HT)-related subclinical hypothyroidism (SH) and no chromosomopathies (Group A) or HT-related SH and Turner syndrome (Group B) or HT-related SH and Down’s syndrome (Group C) (according to the results of Reference 26 study)

Finally, another factor which might exert a negative impact on the long-term evolution of SH is the association with either TS or DS, two chromosomopathies that may be linked with an increased risk of thyroid status deterioration over time [25, 26, 34]. In fact, the prevalence of euthyroidism in two cohorts of children with HT-related SH and either DS or TS was found to be significantly lower, at the end of a 5-year follow-up, than that detected in children with HT-related SH but without DS or TS (Fig. 2).

### Long-term prognosis of thyroid function in the cases presenting with overt hyperthyroidism

Such a presentation pattern of HT, that is also called Hashitoxicosis (Htx), is not very common in the pediatric age, although Htx is generally reported as the second commonest cause of hyperthyroidism in childhood, after GD [35]. This condition is believed to result from unregulated release of stored thyroid hormones, during inflammatory-mediated destruction of thyroid [36]. Presenting symptoms are very similar to those of GD and differential diagnosis between these two conditions may be challenging, if only based on clinical and biochemical findings [36, 37]. Nevertheless, thyrotropin receptor autoantibodies, which are accepted to be one of GD hallmarks [36], are generally absent in the children with Htx [37].

According to the few available reports on the natural history of Htx in pediatric age [36, 37], the hyperthyroid phase is always followed by definitive resolution, with persistent euthyroidism and no hyperthyroid relapses [37]. Management of children with Htx requires only a prolonged clinical and biochemical follow-up, but pharmacological treatment may be occasionally needed only in few selected cases; non-pharmacological therapies are never required [37]. A spontaneous and definitive resolution of hyperthyroidism generally occurs on average five months after Htx presentation, although there is a wide variability between the different individuals (Table 2). The children with a more severe Htx course and a longer duration of the hyperthyroid phase have been reported to exhibit higher thyroid autoantibodies at Htx diagnosis [37]. Nevertheless, such a finding needs to be confirmed by further reports.

**Table 2:** Spontaneous duration of TSH suppression and follow-up periods after TSH normalization (months) in two groups of untreated children with Hashimoto’s thyroiditis who initially presented with either overt (Group A) or subclinical hyperthyroidism (Group B) (according to the results of Reference [37] and [39] studies)

	Group A (Nos. 10)	Group B (Nos. 11)	p
TSH suppression duration			
Mean ± SD	4.8 ± 2.0	7.8 ± 7.1	0.203
Range	3–23	1–24	
Follow-up periods			
Mean ± SD	21.5 ± 11.0	33.6 ± 19.5	0.094
Range	12–39	12–69	

### Long-term prognosis of thyroid function in the cases presenting with subclinical hyperthyroidism

Subclinical hyperthyroidism is defined as a serum TSH concentration below the lower limit of the reference range, when FT4 levels are within their reference ranges [38]. Such a biochemical condition may be detected in 3% of the children at HT presentation, a prevalence that is very close to the one of Htx, i.e. 3.5% [4].

The natural history of HT-related subclinical hyperthyroidism has been so far investigated in only few children [39]. According to the results of that prospective study, HT-related subclinical hyperthyroidism may spontaneously resolve in all cases within the first 1–24 months after HT presentation (Table 2).

On the basis of those findings, it was argued that, at least in childhood, the frequency with which HT-related subclinical hyperthyroidism risks progressing to overt hyperthyroidism should be considered very low, irrespectively of both TSH and FT4 serum concentrations at HT diagnosis [39]. According to other reports, the risk of progression to frank hyperthyroidism in the patients with this biochemical condition is distinctly higher, particularly in the cases with undetectable TSH levels at entry [40–42]. However, it has to be considered that, in those reports, natural history of subclinical hyperthyroidism was investigated in elderly patients [40–42], as against as in the study by Aversa et al. [39], which included only children and adolescents.

According to the results of the only available pediatric study on the natural history of subclinical hyperthyroidism, the evolution of this condition over time does not seem to differ from that observed in the children with Htx, at least in terms of duration of TSH suppression periods. These findings suggest that Htx and HT-related subclinical hyperthyroidism might be simply different stages along the same continuum [39].

### Long-term prognosis of thyroid function in the cases presenting with overt hypothyroidism

A picture of overt hypothyroidism may be observed, at diagnosis of HT, in 22.2% of the children, which represents the 2nd most frequent thyroid function pattern at HT presentation in pediatric age, after euthyroidism [4].

Nevertheless, the natural history of frank hypothyroidism in the children with HT has never been investigated to now, since in these cases a replacement treatment is always initiated immediately after diagnosis [15]. Treatment of overt thyroid failure, in fact, is always mandatory and urgent, especially in very young infants, who are also exposed to the risk of a permanent impairment of neuropsychological development, if not treated early [43].

However, the long-term evolution of thyroid function in the children with HT and overt hypothyroidism might be postulated to be not very far from the one historically reported by Rallison et al. in a large series of children with HT [44].

Finally, when we consider the very different biochemical patterns of thyroid function which may characterize HT presentation in children and adolescents, we should take into account a possible role of environmental factors, such as iodine status. In fact, a hypothyroid presentation pattern may be observed more frequently in iodine deficient areas, whilst a hyperthyroid presentation picture may be found more often in areas where iodine intake is elevated [45, 46]. It has to be clarified, however, that all the patients of our cohort with either overt or subclinical hyperthyroidism at HT presentation exhibited an optimal iodine intake [37, 39].

## Conclusions

a) The long-term evolution of thyroid function in children and adolescents with HT is significantly different according to whether HT has initially presented with either euthyroidism or SH or overt hyperthyroidism or subclinical hyperthyroidism; b) a progressive deterioration of thyroid status over time occurs especially in the children presenting with SH, but may also be observed in those presenting with euthyroidism; c) however, many children with either euthyroidism or SH at entry may be found to be euthyroid even five years after HT presentation; d) a definitive resolution of hyperthyroidism is generally observed within 24 months after HT diagnosis in both the groups of patients with either Htx or subclinical hyperthyroidism at entry.

## Abbreviations

AITDs: Autoimmune thyroid disorders
DS: Down syndrome
GD: Graves’ disease
HT: Hashimoto’s thyroiditis
Htx: Hashitoxicosis
SH: Subclinical hypothyroidism
TS: Turner syndrome

## References

[1] Akamizu T, Amino N, DeGroot LJ, De Groot LJ, Beck-Peccoz P, Chrousos G, Dungan K, Grossman A, Hershman JM, Koch C, McLachlan R, New M, Rebar R, Singer F, Vinik A, Weickert MO (2013). Hashimoto’s Thyroiditis.

[2] Wasniewska M, Vigone MC, Cappa M, Aversa T, Rubino M, De Luca F (2007). Acute suppurative thyroiditis in childhood: relative frequency among thyroid inflammatory diseases. J Endocrinol Investig.

[3] Kabelitz M, Liesenkötter KP, Stach B, Willgerodt H, Stäblein W, Singendonk W (2003). The prevalence of anti-thyroid peroxidase antibodies and autoimmune thyroiditis in children and adolescents in an iodine replete area. Eur J Endocrinol.

[4] Wasniewska M, Corrias A, Salerno M, Mussa A, Capalbo D, Messina MF (2012). Thyroid function patterns at Hashimoto's thyroiditis presentation in childhood and adolescence are mainly conditioned by patients’ age. Horm Res Paediatr..

[5] De Luca F, Santucci S, Corica D, Pitrolo E, Romeo M, Aversa T (2013). Hashimoto’s thyroiditis in childhood: presentation modes and evolution over time. Ital J Pediatr.

[6] De Luca F, Aversa T, Salzano G, Zirilli G, Sferlazzas C, Wasniewska M, Bona G, De Luca F, Monzani A (2015). Autoimmune thyroiditis. Thyroid diseases in childhood: Recent advances from basic science to clinical practice.

[7] Wasniewska M, Corrias A, Arrigo T, Lombardo F, Salerno M, Mussa A (2010). Frequency of Hashimoto’s thyroiditis antecedents in the history of children and adolescents with graves’ disease. Horm Res Paediatr..

[8] Champion B, Gopinath B, Ma G, El-Kaissi S, Wall JR (2008). Conversion to Graves’ hyperthyroidism in a patient with hypothyroidism due to Hashimoto’s thyroiditis documented by real-time thyroid ultrasonography. Thyroid.

[9] Ludgate M, Emerson CH (2008). Metamorphic thyroid autoimmunity. Thyroid.

[10] Troisi A, Novati P, Sali L, Colzani M, Monti G, Cardillo C (2013). Graves’ thyrotoxicosis following Hashimoto’s thyroiditis. Res Rep Endocr Disord.

[11] Zak T, Noczyńska A, Wasikowa R, Zaleska-Dorobisz U, Golenko A (2005). Chronic autoimmune thyroid disease in children and adolescents in the years 1999-2004 in lower Silesia, Poland. Hormones (Athens).

[12] Svensson J, Ericsson UB, Nilsson P, Olsson C, Jonsson B, Lindberg B (2006). Levothyroxine treatment reduces thyroid size in children and adolescents with chronic autoimmune thyroiditis. J Clin Endocrinol Metab.

[13] Demirbilek H, Kandemir N, Gonc EN, Ozon A, Alikasifoglu A (2009). Assessment of thyroid function during the long course of Hashimoto's thyroiditis in children and adolescents. Clin Endocrinol.

[14] Gopalakrishnan S, Chugh PK, Chhillar M, Ambardar VK, Sahoo M, Sankar R (2008). Goitrous autoimmune thyroiditis in a pediatric population: a longitudinal study. Pediatrics.

[15] de Vries L, Bulvik S, Phillip M (2009). Chronic autoimmune thyroiditis in children and adolescents: at presentation and during long-term follow-up. Arch Dis Child.

[16] Özen S, Berk Ö, Şimşek DG, Darcan S (2011). Clinical course of Hashimoto's thyroiditis and effects of levothyroxine therapy on the clinical course of the disease in children and adolescents. J Clin Res Pediatr Endocrinol..

[17] Skarpa V, Kousta E, Tertipi A, Anyfandakis K, Vakaki M, Dolianiti M (2011). Epidemiological characteristics of children with autoimmune thyroid disease. Hormones (Athens).

[18] Radetti G, Gottardi E, Bona G, Corrias A, Salardi S, Loche S (2006). The natural history of euthyroid Hashimoto’s thyroiditis in children. J Pediatr.

[19] Radetti G, Maselli M, Buzi F, Corrias A, Mussa A, Cambiaso P (2012). The natural history of the normal/mild elevated TSH serum levels in children and adolescents with Hashimoto's thyroiditis and isolated hyperthyrotropinaemia: a 3-year follow-up. Clin Endocrinol.

[20] Aversa T, Corrias A, Salerno M, Tessaris D, Di Mase R, Valenzise M (2016). Five-year prospective evaluation of thyroid function test evolution in children with Hashimoto's Thyroiditis presenting with either Euthyroidism or subclinical hypothyroidism. Thyroid.

[21] Vanderpump MP, Tunbridge WM (2002). Epidemiology and prevention of clinical and subclinical hypothyroidism. Thyroid.

[22] De Luca F, Corrias A, Salerno M, Wasniewska M, Gastaldi R, Cassio A (2010). Peculiarities of graves’ disease in children and adolescents with Down’s syndrome. Eur J Endocrinol.

[23] Aversa T, Lombardo F, Corrias A, Salerno M, De Luca F, Wasniewska M (2014). In young patients with turner or down syndrome, Graves’ disease presentation is often preceded by Hashimoto’s thyroiditis. Thyroid.

[24] Valenzise M, Aversa T, Corrias A, Mazzanti L, Cappa M, Ubertini G (2014). Epidemiology, presentation and long-term evolution of graves’ disease in children, adolescents and young adults with turner syndrome. Horm Res Paediatr..

[25] Aversa T, Lombardo F, Valenzise M, Messina MF, Sferlazzas C, Salzano G (2015). Peculiarities of autoimmune thyroid diseases in children with turner or down syndrome: an overview. Ital J Pediatr.

[26] Wasniewska M, Aversa T, Salerno M, Corrias A, Messina MF, Mussa A (2015). Five-year prospective evaluation of thyroid function in girls with subclinical mild hypothyroidism of different etiology. Eur J Endocrinol.

[27] Aversa T, Valenzise M, Salerno M, Corrias A, Iughetti L, Radetti G (2015). Metamorphic thyroid autoimmunity in down syndrome: from Hashimoto’s thyroiditis to graves’ disease and beyond. Ital J Pediatr.

[28] Monzani A, Prodam F, Rapa A, Moia S, Agarla V, Bellone S (2012). Endocrine disorders in childhood and adolescence. Natural history of subclinical hypothyroidism in children and adolescents and potential effects of replacement therapy: a review. Eur J Endocrinol.

[29] Bona G, Prodam F, Monzani A (2013). Subclinical hypothyroidism in children: natural history and when to treat. J Clin Res Pediatr Endocrinol..

[30] Lazarus J, Brown RS, Daumerie C, Hubalewska-Dydejczyk A, Negro R, Vaidya B (2014). European thyroid association guidelines for the management of subclinical hypothyroidism in pregnancy and in children. Eur Thyroid J.

[31] Aversa T, Valenzise M, Corrias A, Salerno M, De Luca F, Mussa A (2015). Underlying Hashimoto's thyroiditis negatively affects the evolution of subclinical hypothyroidism in children irrespective of other concomitant risk factors. Thyroid.

[32] Lazar L, Frumkin RB, Battat E, Lebenthal Y, Phillip M, Meyerovitch J (2009). Natural history of thyroid function tests over 5 years in a large pediatric cohort. J Clin Endocrinol Metab.

[33] Wasniewska M, Corrias A, Aversa T, Valenzise M, Mussa A, De Martino L (2012). Comparative evaluation of therapy with L-thyroxine versus no treatment in children with idiopathic and mild subclinical hypothyroidism. Horm Res Paediatr..

[34] Aversa T, Messina MF, Mazzanti L, Salerno M, Mussa A, Faienza MF (2015). The association with turner syndrome significantly affects the course of Hashimoto’s thyroiditis in children, irrespective of karyotype. Endocrine.

[35] Williamson S, Greene SA (2010). Incidence of thyrotoxicosis in childhood: a national population based study in the UK and Ireland. Clin Endocrinol.

[36] Nabhan ZM, Kreher NC, Eugster EA (2005). Hashitoxicosis in children: clinical features and natural history. J Pediatr.

[37] Wasniewska M, Corrias A, Salerno M, Lombardo F, Aversa T, Mussa A (2012). Outcomes of children with hashitoxicosis. Horm Res Paediatr.

[38] Donangelo I, Braunstein GD (2011). Update on subclinical hyperthyroidism. Am Fam Physician.

[39] Aversa T, Valenzise M, Corrias A, Salerno M, Mussa A, Capalbo D (2014). Subclinical hyperthyroidism when presenting as initial manifestation of juvenile Hashimoto's thyroiditis: first report on its natural history. J Endocrinol Investig.

[40] Rosario PW (2008). The natural history of subclinical hyperthyroidism in patients below the age of 65 years. Clin Endocrinol.

[41] Diez JJ, Iglesias P (2009). An analysis of the natural course of subclinical hyperthyroidism. Am J Med Sci.

[42] Rosario PW (2010). Natural history of subclinical hyperthyroidism in elderly patients with TSH between 0.1 and 0.4 mIU/l: a prospective study. Clin Endocrinol.

[43] Foley TP, Abbassi V, Copeland KC, Draznin MB (1994). Brief report: hypothyroidism caused by chronic autoimmune thyroiditis in very young infants. N Engl J Med.

[44] Rallison ML, Dobyns BM, Keating FR, Rall JE, Tyler FH (1975). Occurrence and natural history of chronic lymphocytic thyroiditis in childhood. J Pediatr.

[45] Laurberg P, Cerqueira C, Ovesen L, Rasmussen LB, Perrild H, Andersen S (2010). Iodine intake as a determinant of thyroid disorders in populations. Best Pract Res Clin Endocrinol Metab.

[46] Ergür AT, Evliyaoğlu O, Şıklar Z, Bilir P, Öcal G, Berberoğlu M (2011). Evaluation of thyroid functions with respect to iodine status and TRH test in chronic autoimmune thyroiditis. J Clin Res Pediatr Endocrinol.

